# Self-immolation and its adverse life-events risk factors: results from an Iranian population 

**DOI:** 10.5249/jivr.v7i1.549

**Published:** 2015-01

**Authors:** Alireza Ahmadi, David C. Schwebel, Shahrzad Bazargan-Hejazi, Kobra Taliee, Hosein Karim, Reza Mohammadi

**Affiliations:** ^*a*^Department of Anesthesiology, Critical Care and Pain Management, Imam Reza Hospital, Kermanshah University of Medical Sciences, Kermanshah, Iran.; ^*b*^Department of Public Health Sciences, Division of Social Medicine, Karolinska Institute, Stockholm, Sweden.; ^*c*^Department of Psychology, University of Alabama at Birmingham, Birmingham, USA.; ^*d*^Department of Psychiatry, College of Medicine, Charles Drew University of Medicine and Science, Los Angeles, CA. USA.; ^*e*^Semel Institute for Neuroscience and Human Behavior, David Geffen School of Medicine, UCLA, CA. USA.; ^*f*^Department of Cardiology, Imam Ali Hospital, Kermanshah University of Medical Sciences, Kermanshah, Iran.

**Keywords:** Case control, Adverse life-events, Self-immolation, Deliberate self-inflicted burns, Suicide, Iran

## Abstract

**Background:**

Despite considerable loss of life by deliberate self-burning in low and middle-income countries, few scholars have examined psychiatric factors such as adverse life events that may be related to self-immolation.

**Methods:**

This case-control study investigated adverse life-events as risk factors for self-immolation patients admitted to a burn center serving the western region of Iran. Variables investigated included the following adverse life-events: unplanned pregnancy, infertility, homelessness, financial hardship, problems with friends, intimate relationship break-up , school or university failure, anxiety about school/university performance, problems at work, personal history of suicide attempts, family history of suicide attempts, individual history of mental disorders, and malignant disease.

**Results:**

Financial hardship (OR=3.35, 95% CI=1.19-9.90), intimate relationship break-up (OR=5.45, 95% CI=1.20-11.99), and personal history of suicide attempts (OR=7.00, 95% CI=1.38-35.48) were associated with increased risk of self-immolation.

**Conclusions:**

This study suggests that financial hardship, intimate relationship break-ups, and personal history of suicide attempts are risk factors for self-immolation. Other variables studied did not play a role as individually protective or risk factors for self-immolation.

Further study is needed to substantiate findings of this study and direct research toward tailoring culturally sensitive, empirically-supported interventions for prevention of self-immolation.

## Introduction

On December 17, 2010, a 26-year-old Tunisian man, Mohamed Bouazizi, doused himself in fuel and lit himself on fire as a protest to financial problems and unemployment. This act became a catalyst for a series of political revolutions in Arab countries.^1^ Bouazizi’s suicide was performed in a manner unfamiliar to many in western countries, but fairly common in Middle Eastern countries. 

Following Bouazizi's self-immolation, several duplicate suicide attempts were documented across the Arab world.^2^ In fact, suicide by deliberate self-burning is quite common in countries like Tunisia, Afghanistan, Iraq, and Iran. What remains unclear to scientists is the factors that may lead individuals to attempt suicide by self-immolation. Knowledge of those factors will play an important role in prevention of this violence act.^3-7^

Iran, where the present research was conducted, has one of the highest reported frequencies of self-immolation in the world. Most self-immolation victims in Iran are young women, and self-burning is the third leading cause of years of life lost (YLL) among women in Iran, after disasters and breast cancer. Studies by our team and others reveal that the most common reasons and risk factors of self-immolation in Iran include spousal conflict, family conflict, inability to adjust or cope with life stressors. ^3,6,8-13^ Survivors of self-immolation, when asked to explain about their reason for attempting self-burning, most typically respond that the attempt was “just a cry for help". ^3,6,8-13^

One area that has received little attention in previous research is the role of adverse life-events among victims of self-burning. This study aimed to investigate the role of adverse life-events in the presentation of self-burning among patients admitted to a regional burn center at Imam Khomeini Hospital in Kermanshah province, in the west of Iran. 

## Methods

**Participants**

Thirty adult patients admitted consecutively to the burn center at Imam Khomeini Hospital, Kermanshah, Iran, following deliberate self-burning were eligible to be enrolled in the study. Patients whose suicide seemed suspicious (i.e., those who denied suicidal intent and for whom there were no corroborating witnesses or data) were excluded. Consecutive patients who met the eligibility criteria were enrolled; all eligible patients agreed to participate (refusal rate= 0%). A control group of 30 individuals was recruited from the community and matched to the patients by living area (district-county, rural/urban), gender, and age. These factors are known risks for self-immolation, so controlling them was deemed important. ^3,6,9^

**Protocol**

Within the first 24 hours of admission to the burn center, we administered the Adverse Life-Event scale with all participants. The Adverse Life-Events scale includes 16 dichotomized items ranging from unplanned pregnancy to having malignant disease (see Table 1).

**Table 1:** Measures of Adverse Life-Events Risk Factors.

• unplanned pregnancy(yes vs. no)
• infertility(yes vs. no)
• homelessness(yes vs. no)
• financial hardship(yes vs. no)
• problems with friends(yes vs. no)
• a relationship break-up (with lover or spouse) (yes vs. no)
• school or university failure(yes vs. no)
• anxiety about school/university performance(yes vs. no)
• problems at work(yes vs. no)
• compulsory marriage(yes vs. no)
• individual history of suicide attempts(yes vs. no)
• sibling or parents history of suicide attempts(yes vs. no)
• individual history of mental disorders(yes vs. no)
• having inability and malignant disease(yes vs. no)

The study protocol was approved by the Kermanshah University of Medical Sciences, Local Research Ethics Committee. Informed consent was obtained from all participants. 

**Data Analysis Plan**

Following consideration of descriptive statistics, the differences between adverse life events in the patient cases versus the control group were considered using chi-square tests (Fisher Exact test was used in the case of small cell sizes). We also performed an independent samples t-test to compare the difference between the mean of adverse events in case and control groups. A p-value ≤ 0.05 and 95% CI was set to identify significant differences.

## Results

Eighty seven percent (87%) of self-burning patients were female, 57% were married, and the mean age was 27 years. Average Total Body Surface Area (TBSA) burned was 60%. Table 2 shows the frequencies of adverse life events across the study groups and Table 3 the results of chi square/Fisher’s test analyses. Three variables emerged as having statistically significant differences between the two groups: financial hardship (x ^2^= 5.41, p = 0.02; OR = 3.45; CI = 1.19-9.90), an intimate relationship break-up (x ^2^= 9.02, p = 0.003; OR = 5.45; CI = 1.20-11.99), and personal history of suicide attempts (x ^2^= 6.67; p = 0.01; OR = 7.00; CI = 1.38-35.48). Individuals who had attempted self-immolation had higher rates of all three adverse events in their history. No other comparisons were statistically significant.

**Table 2:** Adverse Life Events Data of Self-immolation Study (case=30; control=30).

Variables	case	control
**Unplanned pregnancy; N (%)**		
Yes	0(0)	1(3)
No	0(0)	29(97)
**Infertility**		
Yes	3(10)	0(0)
No	27(90)	30(100)
**Homelessness **		
Yes	1(3)	0(0)
No	29(90)	30(100)
**Financial hardship**		
Yes	10(33)	19(63)
No	20(67)	11(37)
**Problems with friends**		
Yes	2(7)	0(0)
No	28(93)	30(100)
**Intimate relationship break-up**		
Yes	20(67)	1(3)
No	10(33)	29(97)
**School/ university failure**		
Yes	3(10)	0(0)
No	27(90)	30(100)
**Anxiety about school/ university performance**		
Yes	25(83)	29(97)
No	5(17)	1(3)
**Problems at work**		
Yes	3(10)	2(7)
No	27(90)	28(93)
Compulsory marriage*		
Yes	1(6)	0(0)
No	16(94)	19(100)
**Personal history of suicide attempts**		
Yes	20(67)	28(93)
No	10(33)	2(7)
**Family (sibling or parents) history of suicide attempts**		
Yes	4(13)	7(23)
No	26(87)	23(77)
**Malignant disease**		
Yes	3(10)	1(3)
No	27(90)	29(97)
**Individual history of mental disorders**		
Yes	3(10)	1(3)
No	27(90)	29(97)

* In married people

**Table 3:** Frequencies and Differences between Self-Immolation and Adverse Life Events (Cases n=30; Controls n=30).

Adverse Life Events	x2	P-value ^a^	Odds Ratio	95% CI
Unplanned pregnancy	0.18	0.54	1.11	0.06-16.76
Infertility	3.16	0.08	1.12	0.23-2.67
Homelessness	1.02	0.31	1.07	0.03-2.79
Financial hardship	5.41	0.02	3.45	1.19-9.90
Problems with friends	2.10	0.15	0.99	0.16-4.58
Intimate relationship break-up	9.02	0.003	5.45	1.20-11.99
School/ university failure	3.16	0.08	1.51	0.13-2.95
Anxiety about school/ university performance	2.96	0.09	4.79	0.75-15.33
Problems at work	0.22	0.64	0.64	0.10-4.15
Compulsory marriage	1.02	0.31	1.00	0.30-3.31
Personal history of suicide attempts	6.67	0.01	7.00	1.38-35.48
Family (sibling or parents) history of suicide attempts	1.00	0.32	1.98	0.51-7.64
Individual history of mental disorders	1.07	0.31	0.31	0.03-3.17
Malignant disease	1.07	0.30	0.31	0.03-3.17

a. Fisher’s exact test is used when N<5

We also considered the difference between the mean numbers of adverse life events reported in both groups. The difference was statistically significant [t (58)=2.68, p = 0.01; M=3.26, SD=1.14 for the cases and M=3.90 , SD=.61 for the controls].

## Discussion

This study revealed that financial hardship, intimate relationship breakup, and a personal history of previous suicide attempts were significant adverse life event risk factors for self-burning. Our results parallel findings in the broader suicide literature. Palacio and colleagues,^14^ for example, used a case-control design with 108 adult suicide attempters and 108 controls matched for age and gender. Those who reported adverse life-events in the last six months, and those who had a family history of suicide, had higher risk of suicide. In another study, Zhang and colleagues^15^ used a matched case-control group of 215 suicide attempters (92 male, 123 female). They reported that hopelessness, negative life-events, and family history of suicide were risk factors of attempted suicide. Way and colleagues^16^ found that the common stressors preceding suicide were inmate-to-inmate conflict, recent disciplinary action, fear, physical illness, and adverse life-events such as loss of good time or disruption of family/friendship relationships in the community. In a more recent study, Krysinska and Lester conducted a meta analysis of 50 suicide related articles.^17^ They found PTSD was a risk factor to increase incidence of past or present suicidality.

Adverse life events may serve as risk factors for suicide across cultural boundaries. Investigators in many countries and cultures across the globe have identified family-related stressors such as unplanned pregnancy, financial hardship, familial tensions, family history of suicide, diagnosed mental disorders, and terminal illness as life stressors associated with suicide risk. ^14,16,18-26^

**New Information from the Present Study**

Our results extend the existing literature in several ways. First, we examined the role of adverse life events on risk for self-immolation rather than for broad risk of suicide. Due to its nature, self-immolation can be attempted impulsively, quickly, and without preparation. It also is more harmful to survivors than other suicide strategies (e.g., poisoning). Thus, risk factors for self-immolation may differ from risk factors for other suicide techniques. Second, we studied risk in the Arab world, a region comparatively understudied in the suicide field. As documented by the case of Mohamed Bouazizi in Tunisia, risk factors and strategies for suicide in the Arab world may be different from those in Western cultures. We found three adverse life events, financial hardship, an intimate relationship break-up, and a personal history of previous suicide attempts, were associated with suicide attempts by self-immolation.

One of our non-significant findings is also worthy of mention. Ten percent of self-immolation cases in our study were infertile, all of them women. Even though infertility was not a statistically significant risk factor (p=0.08), the result may be important given cultural issues. Iranian culture assigns high value to fertility, and the burden of stigmatization is high for infertile women.^27^ A large portion of infertile couples (upwards of 80% in some studies) report anxiety, loss of self-esteem, sexual problems, emotional agony, guilt, marital difficulties, and depression.^28,29^ Others have compared infertility-related psychological problems with that of cancer, ischemic heart disease, and hypertension.^30^ Primary care physicians should consider screening infertile women for undetected signs of suicidal behaviors.

**Prevention Strategies**

The results of this study suggest financial hardship, break-up of an intimate relationship, and a personal history of previous suicide attempts are risk factors for self-immolation in Western Iran. Intervention strategies both to prevent adverse life-events and to counsel at-risk individuals concerning ways to cope with and overcome suicidal thoughts following an adverse event should be developed, tested, and implemented. Culturally sensitive interventions should be used, both at the individual and community levels. These interventions might take a community participatory approach to involve key community members for their suggestions. Research has suggested that the strength of the local people and their networks of relationships help to build trust and mutual commitment for implementation of prevention programs.^31^ Further, local community-action groups and non-government organizations (NGOs) are important entities for self-immolation prevention activities.^32^

**Limitations**

This research had both strengths and limitations. Numerous studies indicate that adverse life-events play important roles in general suicidal attempts and death,^14,16,20,24^ but very few have used case-control designs. Furthermore, no previous data were from developing countries, from Arab cultures, or from self-immolation patients.^25,26,31,33^

Despite these strengths, case-control studies also have limitations. In this study, the control group was selected from the same community and matched by age and gender to consecutive-referral cases to obtain accurate matching, but generalizability is still problematic. It is unclear if these results will generalize to other regions of Iran, to other Arab nations, or to countries with other cultural practices. Our sample was also smaller than desirable, limiting statistical power to detect differences.

We view this work to be a pilot study from which further investigation is warranted. Suicide is a difficult behavior to study in Iran and many other Arab nations, where suicide is considered to be taboo and stigmatized, condemned for religious reasons, and even prosecuted as a criminal offense in some Arab nations.^6^ For these reasons, many victims and victims’ families hide details about suicide. Therefore, research like this study, which includes anonymous interviews with surviving victims of suicide, offers valuable information for treatment and prevention programs.^34^

## Conclusion

This paper presents a study of suicide from a region seldom seen in the Western literature and where suicide is heavily stigmatized. The aim of the study was to describe a case-control comparison of self-inflicted burn victims from a burn center in Iran vs. community controls. The subjects were asked about presence or absence of adverse life events. Findings suggest three types of adverse events, financial hardship, break-up of an intimate relationship, and a personal history of previous suicide attempts are significant risk factors for self-immolation. This finding is in line with the existing suicide literature,^14,15,17^ but extends results to an understudied culture and an understudied suicide strategy.
